# Climatic Changes and Orogeneses in the Late Miocene of Eurasia: The Main Triggers of an Expansion at a Continental Scale?

**DOI:** 10.3389/fpls.2018.01400

**Published:** 2018-09-25

**Authors:** Dong-Rui Jia, Igor V. Bartish

**Affiliations:** ^1^School of Ecology and Environmental Science & Yunnan Key Laboratory for Plateau Mountain Ecology and Restoration of Degraded Environments, Yunnan University, Kunming, China; ^2^Department of Botany, Faculty of Science, Charles University in Prague, Prague, Czechia; ^3^Department of Genetic Ecology, Institute of Botany of the Czech Academy of Sciences, Pruhonice, Czechia

**Keywords:** Asian mountains, ecological opportunity, long-distance dispersal, molecular dating, range expansion, Rosales, vicariance

## Abstract

Migrations from the Qinghai-Tibetan Plateau (QTP) to other temperate regions represent one of the main biogeographical patterns for the Northern Hemisphere. However, the ages and routes of these migrations are largely not known. We aimed to reconstruct a well-resolved and dated phylogeny of *Hippophae* L. (Elaeagnaceae) and test hypothesis of a westward migration of this plant out of the QTP across Eurasian mountains in the Miocene. We produced two data matrices of five chloroplast DNA (cpDNA) and five nuclear DNA markers for all distinct taxa of *Hippophae*. These matrices were used to reconstruct phylogenetic relationships in the genus. In dating analyses, we first estimated the stem node age of Elaeagnaceae using five fossil records evenly distributed across a tree of Rosales. We used this estimate and two fossil records to calibrate the cpDNA and nDNA phylogenies of *Hippophae*. The same phylogenies were used to reconstruct ancestral areas within the genus. The monophyly of *Hippophae*, all five *species*, and most of subspecies was strongly supported by both plastid and nuclear data sets. Diversification of *Hippophae* likely started in central Himalayas/southern Tibet in the early Miocene and all extant distinct species had probably originated by the middle Miocene. Diversification of *Hippophae rhamnoides* likely started in the late Miocene east of the QTP from where this species rapidly expanded to central and western Eurasia. Our findings highlight the impact of different stages in uplift of the QTP and Eurasian mountains and climatic changes in the Neogene on diversification and range shifts in the highland flora on the continent. The results provide support to the idea of an immigration route for some European highland plants from their ancestral areas on the QTP across central and western mountain ranges of Eurasia in the late Miocene.

## Introduction

The Neogene geological processes and climatic changes had tremendous impact on the evolution of biota in different regions of Eurasia (e.g., Comes and Kadereit, 2003; Hewitt, 2004). The Qinghai-Tibetan Plateau (QTP) was a central part of these processes (Tang and Shen, 1996; Zheng et al., 2000; An et al., 2001; Li et al., 2001; Guo et al., 2002; Molnar, 2005). It is also one of the most important global biodiversity and evolutionary hot spots (Myers et al., 2000; Mittermeier et al., 2005; López-Pujol et al., 2011). Recent phylogenetic analyses suggest that many genera of the Northern Hemisphere (NH) temperate plants with the highest diversity in the highlands of Asia originated in the QTP and adjacent regions, and then expanded their ranges to other NH regions (e.g., Wang Y.J. et al., 2009; Zhang et al., 2009; Gao et al., 2013; Roquet et al., 2013). These studies support in general the hypothesis that the QTP has served for long as a major source of migrants to west Eurasia and a craddle of diversification (Wulff, 1943; Axelrod et al., 1996; Comes and Kadereit, 2003). However, ages of disjunction and migration from the QTP to west Eurasia for different ecological groups are not clear yet. Few phylogenetic analyses of taxa with Eurasian ranges have been reported (for a recent review, see Kadereit et al., 2008) and even less studies used dated phylogenies and explicit ancestral area reconstructions for these taxa. Lack of detailed information on direction and ages of migrations in Eurasia, thus prevents studying impact of changes in geology and climate on evolution of different ecological groups of Eurasian flora.

**FIGURE 1:**
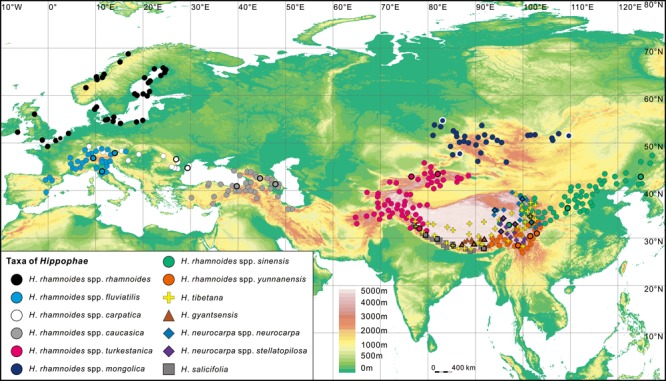
Geographic distribution range of *Hippophae* L. (see Materials and Methods) and the sampling sites (thick outlines; details in **Table 1**).

Ecology and distribution of *Hippophae* L. are highly relevant to the problem of migrations of NH highland plants and their evolutionary responses to the Neogene orogeneses and climatic changes across Eurasia. This shrub or small tree is dioecious, wind-pollinated, and fruits of most species in the genus are juicy favoring long-distance dispersal by birds (Rousi, 1971; Lian et al., 1998). Roots of *Hippophae* form nitrogen-fixing nodules (Bond et al., 1956) and possess an efficient dual symbiosis with mycorrhiza and *Frankia* (Tian et al., 2002). The symbiosis enables *Hippophae* to colonize infertile and bare soil after disturbance (e.g., landslides, flash floods, and dune migration), stabilizing riverbanks, steep slopes, and dunes (Bartish and Swenson, 2004; Bartish, 2016). These plants are therefore early-successional pioneers with habitat engineering function for their communities. *Hippophae* can also serve as a representative of Eurasian distribution and east-central-west disjunctions for mountainous plants (Rousi, 1971; Hyvönen, 1996; Jia et al., 2012). According to the latest detailed revision of the genus by Swenson and Bartish (2002), *Hippophae* includes 15 infraspecific taxa in seven species, two of which are putative hybrids with uncertain taxonomic status (*H. goniocarpa* and *H. litangensis*). All species in the genus are restricted to the QTP region and adjacent areas, except for *H. rhamnoides* that is distributed widely but fragmentally in Asia and Europe (see **Figure 1**). Based on floristic, paleobotanical, and geological inferences, Bobrov (1962) suggested east to west migrations of *Myricaria germanica* (L.) Desv. (Tamaricaceae) and *H. rhamnoides* L. along Eurasian mountain ranges in the Sarmatian, which corresponds to a geological age from the middle to late Miocene (Berggren and Van Couvering, 1974). In contrast, using phylogenetic analyses of morphological characters Hyvönen (1996) suggested a west to east migration followed by range fragmentation and vicariance. Jia et al. (2012) were the first to reconstruct ancestral areas and migration routes for all taxa in *H. rhamnoides* and found support for the hypothesis of the east QTP origin and westward migration. They also estimated ages of diversification within *H. rhamnoides* and suggested that it diversified mostly in the Quaternary. However, this study lacked sufficient power to resolve the relationships among the western subspecies; and its age estimates relied exclusively on secondary calibrations, which can result in considerable age underestimates (Sauquet et al., 2012). The oldest fossil records of *H. rhamnoides* for long were known only from the late Pleistocene (Lang, 1994). Nevertheless, in a recent study, fossil pollen grains of the species that range in age from the late Miocene to the early Pleistocene were reported from several localities in southern Europe (Biltekin, 2010). In view of these latest advances in paleobotany, it becomes obvious that ages of diversifications within the genus, and of the putative migration from the east QTP to west Eurasia should be reconsidered. Given the range and ecological characteristics of *Hippophae*, we assume that application of robust phylogenetic reconstructions and accurate dating across the whole genus can provide useful insights into floristic turnovers on the continent in the Neogene.

Here, we report molecular phylogenetic, fossil-calibrated dating, and ancestral area reconstructions in a thorough sample of all distinct taxa (species/subspecies) of *Hippophae* accepted by Swenson and Bartish (2002). We sampled three populations per taxon across its range, and five DNA fragments each from chloroplast and nuclear genome. Due to the lack of fossil records and unkonwn age of the stem node of *Hippophae*, we performed the molecular dating analysis in two stages. The first used five deep fossil calibration points in a multi-gene data set including all genera of Elaeagnaceae and all families of Rosales. The second used chloroplast and nuclear sequences of *Hippophae*, two fossils, and a secondary calibration point of Elaeagnaceae stem node derived from the first stage. We believe this sampling effort and dating strategy are both necessary and sufficient to answer the following questions. (1) When and where did all distinct taxa in *Hippophae* originate? (2) When and how exactly did the evolutionary processes in these taxa result in their current disjunct ranges across Eurasia from East Asia to Europe? We discuss later how answering these questions can contribute to a better understanding of the impact of the Neogene orogeneses and climatic changes on evolutionary processes in biota of Eurasian highlands.

## Materials and Methods

### Data Set of Rosales

We used an earlier published multi-gene data set of 25 Rosales taxa (Zhang et al., 2011) and extended it by including six more species (*Hippophae tibetana* and *salicifolia, Shepherdia argentea* and *canadensis*, and *Elaeagnus angustifolia* and *umbellata*) from Elaeagnaceae and one more plastid region (*trn*L-F). The final data set comprised 11 plastid (*rbc*L, *atp*B, *mat*K, the *psb*BTNH region (=4 genes), *rpo*C2, *ndh*F, *rps*4, and *trn*L-F) and two nuclear genes (18S and 26S nuclear ribosomal DNA) for 31 species of Rosales. Individual gene alignments were concatenated into a supermatrix of characters with unsampled values coded as missing data. We chose *Begonia* (Begoniaceae) as outgroup to root the tree of Rosales based on Zhang et al. (2011). Details of GenBank accession numbers are listed in **Supplementary Table S1**.

### Data Sets of *Hippophae* L.

Based on the key of Swenson and Bartish (2002) and geographic information, we sampled three populations for each distinct taxon of the genus *Hippophae*. This sampling design aimed to obtain representatives across whole ranges and/or main lineages of each taxon, and to allow us robust tests of reciprocal monophyly and ancestral area reconstructions for all taxa. Geographic representation of the main lineages within the following taxa was inferred from the literature: *H. tibetana* (Wang et al., 2010; Jia et al., 2011), *H. neurocarpa* (Kou et al., 2013), *H. gyantsensis* (Jia et al., 2016), and *H. rhamnoides* ssp. *yunnanensis* and *sinensis* (Jia et al., 2012). Within *H. salicifolia* and several subspecies of *H. rhamnoides* (ssp. *caucasica, fluviatilis, mongolica*, and *turkestanica*), phylogeographic relationships have not yet been resolved using informative sequences from a comprehensive sample of populations. We therefore selected three representative populations across geographic range of each of these taxa. Finally, *H. rhamnoides* ssp. *carpatica* and *rhamnoides* were shown to be closely related with a broad sharing of the same cpDNA haplotype among multiple populations (Bartish et al., 2002, 2006), we thus used three populations to represent the two subspecies across their joint range. Three species from the other two genera of the family Elaeagnaceae (*Elaeagnus triflora, E. umbellata*, and *Shepherdia argentea*) were also used in our phylogenetic and dating analyses on *Hippophae*. Included materials, voucher information and sources are listed in **Table 1**.

**Table 1:** Sampling information of *Hippophae* L. Three species from *Shepherdia* Nutt. and *Elaeagnus* L. were included.

Taxon	Population	Herbarium	Voucher	Location	Collector	Year of collection
*H. salicifolia*	4079	IBP	PRA-00004079	Hildum, Humla, Nepal	R. Maan	2009
	09XZ040	LZU	LiuJQ-09XZ-LZT-040	Gongri, Xizang, China	J-Q. Liu	2009
	P	AMU	P	Chamoli, Uttaranchal, India	S. Raina	2011
*H. gyantsensis*	06252	LZU	Liu-06252	Tingri, Xizang, China	J-Q. Liu	2006
	06217	LZU	Liu-06217	Khangmar, Xizang, China	J-Q. Liu	2006
	2568	LZU	2568	Lhasa, Xizang, China	J-Q. Liu	2004
*H. neurocarpa*						
ssp. *neurocarpa*	YNG1	LZU	YNG1/4	Golmud, Qinghai, China	J-Q. Liu	2003
	1522	LZU	1522	Qilian, Qinghai, China	J-Q. Liu	2003
	MM-31	LZU	MM-31	Jiuzhi, Qinghai, China	J-Q. Liu	2007
ssp. *stellatopilosa*	Ao129	LZU	Ao129	Dingqing, Xizang, China	J-Q. Liu	2005
	QML1	LZU	QML1-2	Qumalai, Qinghai, China	J-Q. Liu	2003
	Ao111	LZU	Ao111	Shiqu, Sichuan, China	J-Q. Liu	2005
*H. tibetana*	07138	LZU	Liu-07138	Jilong, Xizang, China	J-Q. Liu	2007
	Henan	LZU	Henan	Henan, Qinghai, China	J.-Q. Liu	2008
	ST	AMU	ST	Spiti, Himachal Pradesh, India	S. Raina	2011
*H. rhamnoides*						
ssp. *yunnanensis*	06309	LZU	Liu-06309	Deqin, Yunnan, China	J-Q. Liu	2006
	06321	LZU	Liu-06321	Daofu, Sichuan, China	J-Q. Liu	2006
	06324	LZU	Liu-06324	Xiaojin, Sichuan, China	J.-Q. Liu	2006
ssp. *sinensis*	Ao96	LZU	Ao96	Yushu, Qinghai, China	J-Q. Liu	2005
	05276	LZU	Liu-05276	Ganquan, Shaanxi, China	J-Q. Liu	2005
	05179	LZU	Liu-05179	Xifeng, Liaoning, China	J-Q. Liu	2005
*H. rhamnoides*						
ssp. *turkestanica*	GL	LZU	GL	Gongliu, Xinjiang, China	J-Q. Liu	2005
	SKZ	AMU	SKZ	Spiti, Himachal Pradesh, India	S. Raina	2011
	12413	S	S08-12413	Almaty, Kazakhstan	L. Stenberg	2008
ssp. *mongolica*	05047	LZU	Liu-05047A	Bu’erjin, Xinjiang, China	J-Q. Liu	2005
	6667	IBP	PRA-00006667	Novokizhiginsk, Buryatiya, Russia	I. Bartish; A. Borisyuk	2012
	6657	IBP	PRA-00006657	Berdsk, Novosibirskaya, Russia	I. Bartish; A. Borisyuk	2012
ssp. *caucasica*	0402	SUA	0402-1-36	Usukhchay, Dagestan, Russia	M. Rabadanov	1998
	21286	S	S09-21286	Kazbegi, Georgia	J. Klakenberg	1993
	9835	SUA	2898492	Hazi Mehmet, Turkey	M. Kucuk	1996
ssp. *fluviatilis*	SW1	LZU	Liu-SW1	Wildhaus, Switzerland	Y-M. Yuan	2005
	6757	IBP	PRA-00006757	Il Mulino, Firenzuola, Italy	I. Bartish; I. Schanzer	2012
	6682	IBP	PRA-00006682	Kirchdorf an der Krems, Austria	I. Bartish; I. Schanzer	2012
ssp. *carpatica*	9897	SUA	9897-13-5	Gheorghe, Romania	P. Mladin	1998
	9898	SUA	9898-13-16	Serpeni, Romania	P. Mladin	1998
ssp. *rhamnoides*	6672	IBP	PRA-00006672	Oostduinkerke, Belgium	F. Verloove	2012
*Elaeagnus triflora*	IVB-29	S	IVB-29	Glen Allyn, Queensland, Australia	I. Bartish; A. Ford	2004
*Elaeagnus umbellata*	07078	LZU	Liu-07078	Jilong, Xizang, China	J-Q. Liu	2007
*Shepherdia argentea*	6777	IBP	PRA-00006777	Canada^a^	P. Sekerka	2011

*Herbarium: IBP, Institute of Botany, Academy of Sciences, Prague, Czech Republic; LZU, Lanzhou University, Lanzhou, China; AMU, Amity University, New Delhi, India; S, Museum of Natural History, Stockholm, Sweden; SUA, Balsgård research station, University of Agricultural Sciences, Alnarp, Sweden. ^*a*^Cultivated in Botanical Garden of Prague City, Troja, Czech Republic. ACCID: 1993.00168, Canada IS 237.*

We used DNeasy^TM^ Tissue Kit (Qiagen, Germany) to isolate total genomic DNA from silica gel dried leaves. We sequenced five chloroplast DNA (cpDNA: *trn*C^GCA^-ycf6, *trn*D^GUC^-*trn*T^GGU^, *trn*L^UAA^-*trn*F^GAA^, *trn*S^UGA^-*trn*fM^CAU^, and *trn*S^GCU^-*trn*G^UCC^) and five nuclear DNA regions (nDNA: *At*103, *G3pdh*, ITS, *Ms*, and *Tpi*) for all taxa. Primers used for amplification and sequencing of these regions are provided in **Supplementary Table S2**. Polymerase chain reactions (PCRs) were performed in 25 μL reaction mixture volumes using reagents and manufacturer’s instruction for *Taq* DNA polymerase (Thermo Scientific, United States) in a Mastercycler (Eppendorf, Germany). PCR products were purified using QIAquick PCR Purification Kit (Qiagen, Germany) and sequencing reactions were conducted with ABI Prism Bigdye^TM^ Terminator v.3.1 Cycle Sequencing Kit (Applied Biosystems, United States). Sequences were obtained using an ABI 3130 Genetic Analyzer (Applied Biosystems). MEGA v.4 (Tamura et al., 2007) was used to align produced sequences and adjust them manually.

Chloroplast and nuclear sequences were concatenated separately to make two data sets. We chose *Rhamnus davurica* (Rhamnaceae) as outgroup to root the tree, according to Zhang et al. (2011) and our results (**Supplementary Figure S1**). Available outgroup sequences were taken from GenBank. Newly generated sequences were deposited in GenBank (**Supplementary Table S3**). GAPCODER (Young and Healy, 2003) was used to edit indels as separate characters for inclusion in Bayesian analyses.

### Phylogenetic Analyses

Partitioned Bayesian and Maximum Likelihood (ML) analyses were conducted on the three concatenated data sets. Bayesian inference was performed in MRBAYES v.3.2.1 (Ronquist and Huelsenbeck, 2003). We determined the best fitting model of sequence evolution for each individual region using the Akaike Information Criterion (Akaike, 1974) as employed in JMODELTEST v.2.1.3 (Darriba et al., 2012). Two independent runs with one cold and three incrementally heated Monte Carlo Markov chains (MCMCs) were run for 5,000,000 generations, with trees sampled every 500th generation. A standard discrete model (Lewis, 2001) was applied to the indel matrix. Model parameters were unlinked across partitions. We discarded the first 2,500 trees out of the 10,001 trees as burn-ins and used the remaining trees to build a 50% majority rule consensus tree.

Maximum likelihood analyses were performed using RAxML v.7.2.7 (Stamatakis, 2006). A separate General Time Reversible + Gamma model (GTR + G) of nucleotide substitution was specified for each data partition, and 500 independent searches were conducted. Support values for nodes in the phylogenetic tree were estimated across 1,000 pseudoreplicates using the GTR + CAT model (Stamatakis et al., 2008) and mapped thereafter onto the best-scoring tree from the 500 independent searches.

### Selection of Fossils for Calibration

**FIGURE 2:**
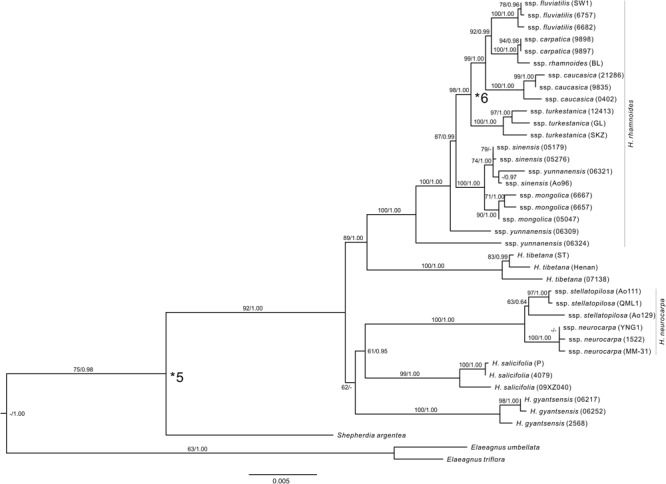
The ML tree of *Hippophae* L. from the combined cpDNA fragments. Support values (ML Bootstrap percentage/Bayesian posterior probability) are provided at nodes. Fossil calibrations were used at nodes indicated with asterisks (see **Supplementary Table S4**).

We constrained the maximum age of the crown node of Rosales using maximum age estimate for this node (103 Ma) from Wang H. et al. (2009). This study was based on a comprehensive sample of families from Rosids (represented by 104 species), sequences of 12 genes (10 cpDNA and two nDNA), and on seven fossil records to calibrate the tree. This sampling effort indicates that estimates of ages of diversification among the sampled orders, including the stem and crown nodes of Rosales, can be fairly robust. We selected from paleobotanical literature five fossil records, which can define the minimum ages of the stem nodes of respective taxa and clades in our tree (**Supplementary Figure S2** and **Supplementary Table S4**). *Celtis aspera* (Newberry) Manchester, Akhmetiev, and Kodrul was selected based on well-preserved endocarps and leaves from numerous localities in North America and Asia in the late Paleocene and leaves throughout the Paleocene in North America (Manchester et al., 2002). We used the late Paleocene records of this fossil species in our analyses to calibrate the stem node of *Celtis*. *Triorites minutipori* Muller was selected based on pollen from Turonian–Senonian (Coniacian) of Malaysia (Muller, 1968), which was considered later as a reliable representative of Ulmaceae (Muller, 1981). This fossil was used therefore to calibrate the stem node of Ulmaceae. *Paliurus clarnensis* Burge & Manchester has a set of morphological apomorphies to assign this fossil species reliably to extant *Paliurus* (Burge and Manchester, 2008). We calibrated by this fossil the stem node of *Hovenia*, which in our sample represents the crown node of Paliureae (Richardson et al., 2000; Islam and Simmons, 2006). Compound drupes of *Ficus* L. were described from the early Eocene London Clay Flora by Chandler (1962) and can be used to calibrate the stem node of *Ficus* in our sample. Leaves of *Shepherdia weaveri* Becker were described from the late Eocene flora of southwestern Montana (Becker, 1960) and recently included into a revised list of paleotaxa from this locality by Lielke et al. (2012), who also provided revised geochronology for the locality. We used this fossil species and age of its geological stratum to calibrate the stem node of *Shepherdia* in our sample. Fossil pollen grains of *H. rhamnoides* from the late Miocene (Melinte-Dobrinescu et al., 2009) of Anatolia and south of the Balkan peninsula document the earliest records of the genus (Biltekin, 2010). The fossil cannot be reliably assigned to any subspecies within *H. rhamnoides* due to the lack of diagnostic characters (Sorsa, 1971). Nevertheless, molecular phylogenetic analyses (Bartish et al., 2000; Jia et al., 2012) and ancestral area reconstructions (Jia et al., 2012) suggested that geographic localities of these records can represent an ancestral area of the strongly supported monophyletic group of the four western subspecies (ssp. *carpatica, caucasica, fluviatilis*, and *rhamnoides*). We used therefore this fossil record to calibrate the minimum age of the stem node of the group (**Figure 2** and **Supplementary Table S4**).

### Divergence Time Estimation in Rosales and *Hippophae*

We used penalized likelihood (PL) and Bayesian inference methods, implemented in R8S v.1.71 (Sanderson, 2002, 2003) and BEAST v.1.7.5 (Drummond et al., 2012), respectively, to infer divergence times in both Rosales and *Hippophae* phylogenies. For PL analyses, the ML trees with branch lengths (phylograms) obtained with RAXML were used. The outgroups were pruned from the trees prior to all analyses. The smoothing parameters (λ) were determined by cross-validation analysis. We used the truncated-Newton (TN) algorithm as recommended for PL in R8S manual and chose the additive penalty over the log penalty due to our balanced calibrations in the trees. All fossil constraints were applied as minimum ages, while secondary calibrations were enforced as maximum age constraints. Confidence intervals for the divergence date estimates were obtained in a bootstrap-based approach. We used the best-scoring ML tree as a fixed topology and estimated sets of branch lengths from 1,000 bootstrap replicates using the software RAXML (Sauquet et al., 2012). Divergence dates were estimated from the resulting 1,000 trees using the software R8S. Settings were as described above, except that the smoothing parameters were fixed to the values obtained for the original data sets.

For Bayesian analyses, two different uncorrelated lognormal (UCLN) relaxed clock models with fossils treated as being drawn from either a uniform distribution (UCLN-uniform) or a lognormal distribution (UCLN-lognormal) were implemented. For uniform priors, fossil constraints were implemented as hard minimum bounds. For lognormal priors, the ages of the fossils were set as offset values with Log (mean) = 0 and Log (SD) = 1. Age ranges for secondary calibrations were all implemented as uniform priors. The optimal model of molecular evolution selected in JMODELTEST was specified for each partition, and a Yule prior was specified for the tree. We initiated MCMC analyses from ultrametric starting tree with branch lengths that satisfied the calibration constraints. We sampled all parameters once every 1,000 steps from 10,000,000 MCMC steps, with the first 25% of samples discarded as burn-in. The program TRACER v.1.5.0 (Rambaut and Drummond, 2007) was used to examine convergence of chains to the stationary distribution. Trees then were compiled into a maximum clade credibility (MCC) tree using TREEANNOTATOR v.1.7.5 (Drummond et al., 2012) to display mean node ages and highest posterior density (HPD) intervals at 95% (upper and lower) for each node.

### Ancestral Area Reconstructions in *Hippophae*

We ran Bayesian dispersal-vicariance analyses (Bayes-DIVA) using RASP v.3.1 (Yu et al., 2015) to infer the biogeographical history of *Hippophae* based on both cpDNA and nDNA data sets. In this analysis, we defined nine regions across the range of *Hippophae* (**Figure 1**; Rousi, 1971; Chinese Virtual Herbarium^1^; Global Biodiversity Information Facility^2^) according to the floristic regionalization of China (Wu et al., 2011) and the world (Takhtajan, 1986): (A) the south QTP (the central Himalayas and southern Tibet); (B) the east QTP (the Hengduan); (C) the north QTP (Tangut); (D) the west QTP (including Tianshan, Pamir, Hindu Kush, and Karakoram); (E) northern China; (F) the Mongolian Plateau (including Altai-Sayan and South Siberian Plain); (G) the Caucasus and Anatolian highlands; (H) Carpathians and Europe north of Alps; and (I) the Alps, Apennines, and Pyrenees. We loaded 10,001 trees previously produced in BEAST and chose the F81 model for the Bayesian MCMC analyses, allowing for different rates of change among ancestral areas. Additionally, for comparative purposes, we implemented the dispersal-extinction-cladogenesis model in RASP v.3.1 as well, with maximum range size set to two.

## Results

### Phylogenetic Relationships of Rosales and Elaeagnaceae

Based on the data set consisting of 18,335 aligned nucleotide sites for the 13 gene partitions (**Supplementary Table S5**), we reconstructed the phylogeny of Rosales. ML and BI approaches yielded an identical topology with slightly different support values (**Supplementary Figure S1**). Within the family Elaeagnaceae, the three genera were each strongly (BP = 100%; PP = 1.00) supported as monophyletic, and *Elaeagnus* were sister to the well-supported (BP = 100%; PP = 1.00) clade of *Hippophae* and *Shepherdia*.

### Phylogenetic Relationships of *Hippophae* Based on cpDNA

The combined cpDNA matrix of *Hippophae* included a total of 5,462 aligned base pairs (**Supplementary Table S6**). The ML analysis with RAXML yielded a well-resolved phylogenetic tree (**Figure 2**). The monophyly of *Hippophae* was strongly supported (BP = 92%). All taxa of *Hippophae* except for *H. rhamnoides* ssp. *sinensis* and *yunnanensis* were recovered as monophyletic with strong statistical support (BP > 90% for all taxa except *H. neurocarpa* ssp. *stellatopilosa* with BP = 63%). *H. gyantsensis, H. salicifolia*, and *H. neurocarpa* formed a clade with a low support (BP = 62%). This clade was sister to the moderately (BP = 89%) supported clade composed of *H. tibetana* and *H. rhamnoides*. Within *H. rhamnoides*, two lineages of ssp. *yunnanensis* were placed basal to the clade including the other taxa. The latter clade consisted of two sub-clades, both receiving strong support (BP > 98%). One of these sub-clades included ssp. *sinensis* and *mongolica*, the second one included ssp. *turkestanica*, and the four other Asia Minor/European subspecies (ssp. *caucasica, carpatica*/*rhamnoides*, and *fluviatilis*, all listed here as ordered in the grade). Tree topology recovered from Bayesian analysis was largely congruent with the ML tree. The main discrepancy regarded the unresolved relationships between *H. gyantsensis* and the other two clades containing *H. neurocarpa*/*H. salicifolia* and *H. tibetana*/*H. rhamnoides*, respectively (**Figure 2**).

### Phylogenetic Relationships of *Hippophae* Based on Nuclear DNA

**FIGURE 3:**
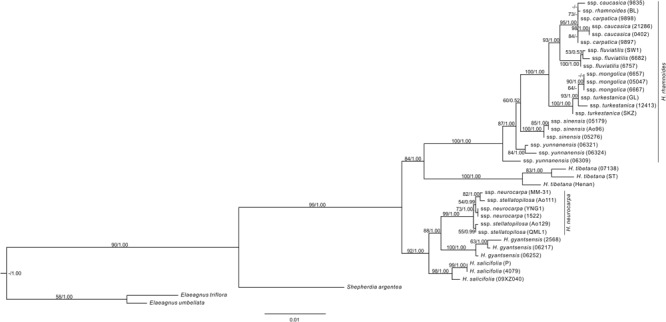
The ML tree of *Hippophae* L. from the combined nDNA fragments. Support values (ML Bootstrap percentage/Bayesian posterior probability) are provided at nodes.

The aligned length of the combined nuclear DNA matrix of *Hippophae* was 2,958 base pairs (**Supplementary Table S6**). The monophyly of *Hippophae* was strongly supported by both the ML and Bayesian methods (BP = 99%, PP = 1.00; **Figure 3**). The clade of *H. gyantsensis, H. neurocarpa*, and *H. salicifolia* received strong support (BP = 92%; PP = 1.00), in which *H. salicifolia* was sister to the sub-clade of the other two taxa, and all species were monophyletic with strong support (BP > 98%; PP = 1.00). *H. tibetana* was placed sister to *H. rhamnoides* in the moderately (BP = 84%) to strongly (PP = 1.00) supported clade where each species is monophyletic with strong support (BP = 100%; PP = 1.00). Within *H. rhamnoides*, we found incongruence with cpDNA phylogenetic placement of *H. rhamnoides* ssp. *mongolica*: it formed a strongly supported (BP = 90%; PP = 1.00) clade within ssp. *turkestanica*. Additionally, monophyly of ssp. *carpatica, caucasica*, and *yunnanensis* was not supported.

### Divergence Time Estimates Within Elaeagnaceae

In general, PL and two Bayesian analyses (performed in MRBAYES v.3.2.1 and in BEAST v.1.7.5) yielded similar estimates of ages with largely overlapping confidence intervals (**Supplementary Tables S7–S9**). We thus report the divergence times estimated under the Bayesian UCLN-lognormal model in the following text. Ages of the stem and crown nodes for Elaeagnaceae were estimated to 89.3 (95% HPD: 85.7–93.0) and 40.6 (95% HPD: 36.9–44.0) Ma, respectively (**Supplementary Figure S2** and **Supplementary Table S7**). We enforced 44.1 Ma (upper 95% HPD interval under the UCLN-uniform model) as maximum age of the crown node of Elaeagnaceae in the dating analyses of *Hippophae*.

### Estimation of Ages and Ancestral Area Reconstructions Within *Hippophae*

**FIGURE 4:**
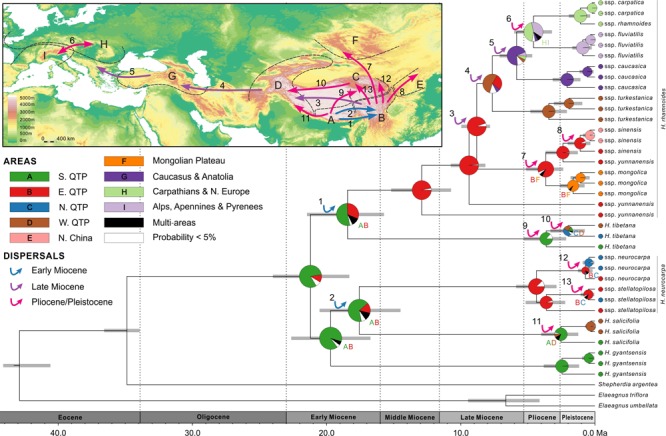
Combined chronogram and biogeographical analysis of *Hippophae* L. based on cpDNA data. Divergence times were derived from Bayesian analysis treating priors on fossils as being drawn from a lognormal distribution. Gray bars represent 95% credible intervals. Node charts show the marginal probabilities of alternative ancestral distributions from Bayesian dispersal-vicariance analysis (Bayes-DIVA) implemented in RASP. Multiple area distributions were labeled with respective colored characters. The detected dispersal events by Bayes-DIVA analysis are shown with colorful arrows on the chronogram and map. See **Table 2** for age and probability estimates of the inferred dispersals.

According to our age estimates based on cpDNA, all species within *Hippophae* had diversified in the early Miocene (21.2–17.6 Ma; **Figure 4** and **Supplementary Table S8**). RASP Bayes-DIVA analyses provided relatively strong evidence that the ancestral area for *Hippophae* was in the south QTP with a marginal probability (MP) of 81.1% (**Figure 4** and **Supplementary Table S8**). The ancestral areas of *H. neurocarpa* and *H. tibetana* were inferred to be the east QTP (MP = 87.4%) and the south QTP (MP = 86.4%), respectively. Additionally, results of our dating analyses suggested that ages of diversification within most taxa in the genus were not older than the late Pliocene or Pleistocene (3.6–0.2 Ma).

Diversification within *H. rhamnoides* started in the middle Miocene and proceeded to the early Pliocene (12.9–3.7 Ma). This species had a high probability (MP = 96.7%) of ancestral area in the east QTP, from where several lineages had dispersed to the west QTP (dispersal 3: 8.8 Ma), north to the Mongolian Plateau (dispersal 7: 3.7 Ma), and northeast to Northern China (dispersal 8: 1.1 Ma) (**Figure 4** and **Table 2**). Our analyses also indicated subsequent westward dispersals out of the west QTP (dispersal 4: 7.6 Ma) and Asia Minor (dispersal 5: 5.8 Ma). Finally, the species dispersed from Carpathians to Alps (dispersal 6: 4.6 Ma). All dispersals were followed by vicariance events, according to the RASP analyses. Additionally, we inferred three local extinction events for ancestors of *H. neurocarpa, H. rhamnoides* ssp. *turkestanica*, and a clade of Asia Minor/European subspecies of *H. rhamnoides*, occurring in the south, east, and west QTP, respectively. We also noted some different biogeographical scenarios at nodes with the lowest relative probabilities. In particular, results of these analyses indicated two roughly contemporaneous dispersals from the west QTP to Asia Minor and Europe, respectively. However, these uncertainties do not influence our main conclusions specified in Discussion.

**Table 2:** Summary statistics of the 13 dispersal events detected in the *Hippophae* cpDNA phylogeny using Bayesian dispersal-vicariance analysis implemented in RASP.

Dispersal events	AAR of parent node				AAR of daughter node				Age of parent node (Ma)		Age of daughter node (Ma)	
	1	MP	2	MP	1	MP	2	MP	Mean	95% HPD	Mean	95% HPD
1	A	(52.7)	B	(31.8)	B	(96.7)	AB	(2.6)	18.4	(15.7–21.5)	12.9	(10.7–15.2)
2	A	(67.0)	B	(15.0)	B	(87.4)	AB	(4.3)	17.6	(14.5–20.5)	4.4	(2.9–5.9)
3	B	(94.4)	BD	(1.4)	D	(53.6)	B	(27.3)	8.8	(7.8–10.0)	7.6	(7.2–8.2)
4	D	(53.6)	B	(27.3)	G	(69.9)	I	(7.4)	7.6	(7.2–8.2)	5.8	(4.7–7.1)
5	G	(69.9)	I	(7.4)	H	(44.2)	I	(37.4)	5.8	(4.7–7.1)	4.6	(3.2–6.0)
6	H	(44.2)	I	(37.4)	I	(98.6)	HI	(1.0)	4.6	(3.2–6.0)	0.9	(0.2–1.7)
7	B	(88.5)	BF	(6.7)	F	(93.3)	BF	(5.6)	3.7	(2.4–5.1)	1.6	(0.8–2.6)
8	B	(93.3)	BE	(4.7)	E	(94.5)	BE	(4.7)	1.1	(0.3–2.0)	0.4	(0.01–0.9)
9	A	(86.4)	B	(2.8)	C	(43.1)	D	(28.6)	3.6	(2.1–5.3)	2.0	(0.8–3.3)
10	C	(43.1)	D	(28.6)	D	–	–	–	2.0	(0.8–3.3)	0	0
11	A	(75.9)	AD	(11.0)	D	(97.4)	AD	(2.2)	2.5	(1.2–4.0)	0.3	(0.003–0.7)
12	B	(79.0)	BC	(11.7)	C	(95.7)	BC	(3.7)	0.7	(0.2–1.3)	0.5	(0.05–0.9)
13	B	(84.6)	BC	(10.8)	C	–	–	–	0.5	(0.05–1.1)	0	0

*Dispersal numbers and area codes refer to **Figure 4**. For ancestral area reconstructions (ARR), the first two areas with the highest marginal probability (MP; in percent) are reported for both the parent and daughter nodes of each dispersal event. Age estimates are derived from Bayesian relaxed clock analysis treating priors on fossils as being drawn from a lognormal distribution. The mean date in million years ago (Ma) and the 95% highest posterior density intervals are given for each parent and daughter node.*

**FIGURE 5:**
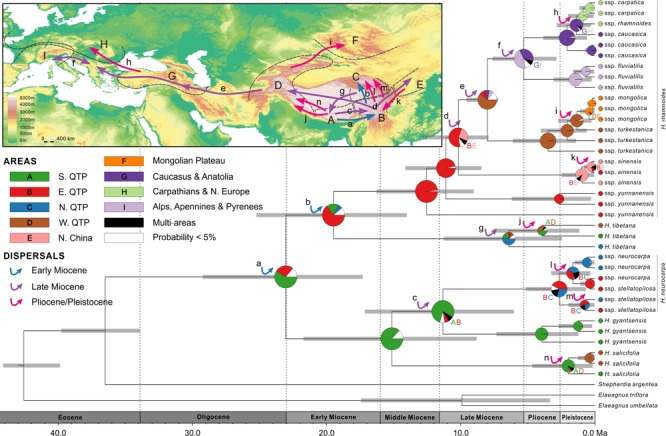
Combined chronogram and biogeographical analysis of *Hippophae* L. based on nDNA data. Divergence times were derived from Bayesian analysis treating priors on fossils as being drawn from a lognormal distribution. Gray bars represent 95% credible intervals. Node charts show the marginal probabilities of alternative ancestral distributions from Bayesian dispersal-vicariance analysis (Bayes-DIVA) implemented in RASP. Multiple area distributions were labeled with respective colored characters. The detected dispersal events by Bayes-DIVA analysis are shown with colorful arrows on the chronogram and map. See **Table 3** for age and probability estimates of the inferred dispersals.

RASP analysis based on nDNA well supported the diversification within *Hippophae* in the early Miocene and its origination in the south QTP (**Figure 5** and **Supplementary Table S9**). The detected 14 dispersal events (dispersals a-n; **Table 3**) were estimated to have occurred in the early Miocene, late Miocene, and the Pliocene/Pleistocene, respectively. However, unlike the reconstructions based on cpDNA, a dispersal from the west QTP to the Mongolian Plateau was indicated instead (dispersal i: 1.3 Ma). Besides, an additional dispersal from the north QTP to the south QTP was detected (dispersal g: 6.6 Ma).

**Table 3:** Summary statistics of the 14 dispersal events detected in the *Hippophae* nDNA phylogeny using Bayesian dispersal-vicariance analysis implemented in RASP.

Dispersal events	AAR of parent node				AAR of daughter node				Age of parent node (Ma)		Age of daughter node (Ma)	
	1	MP	2	MP	1	MP	2	MP	Mean	95% HPD	Mean	95% HPD
a	A	(58.7)	B	(27.1)	B	(65.3)	A	(16.9)	22.9	(17.1–28.4)	18.9	(13.7–24.5)
b	B	(65.3)	A	(16.9)	C	(53.0)	A	(20.8)	18.9	(13.7–24.5)	6.6	(2.3–11.9)
c	A	(78.5)	AB	(7.6)	B	(41.7)	C	(30.3)	11.6	(6.1–17.7)	2.9	(0.8–5.7)
d	B	(58.3)	E	(25.6)	E	(84.5)	BE	(11.2)	10.2	(8.0–12.6)	1.1	(0.1–2.7)
					D	(61.1)	< 5%	–			7.9	(7.2–8.9)
e	D	(61.1)	< 5%	–	I	(45.5)	G	(36.2)	7.9	(7.2–8.9)	5.3	(3.1–7.6)
f	I	(45.5)	G	(36.2)	G	(96.7)	<5%	–	5.3	(3.1–7.6)	2.2	(0.5–4.0)
g	C	(53.0)	A	(20.8)	A	(49.2)	D	(33.0)	6.6	(2.3–11.9)	3.6	(0.9–7.4)
h	G	(90.2)	GH	(5.9)	H	(94.7)	<5%	–	1.5	(0.3–3.1)	0.9	(0.04–2.1)
i	D	(95.4)	<5%	–	F	(93.5)	DF	(5.5)	1.3	(0.2–2.7)	0.4	(0.01–1.1)
j	A	(49.2)	D	(33.0)	D	–	–	–	3.6	(0.9–7.4)	0	0
k	E	(71.7)	BE	(17.8)	B	–	–	–	0.3	(0–0.8)	0	0
l	B	(38.6)	C	(36.0)	C	(96.2)	<5%	–	1.8	(0.4–3.7)	0.4	(0–1.2)
m	B	(45.4)	BC	(30.3)	C	–	–	–	1.3	(0.1–2.8)	0	0
n	A	(81.6)	AD	(9.1)	D	(96.9)	<5%	–	2.3	(0.2–5.5)	0.4	(0–1.4)

*Dispersal numbers and area codes refer to **Figure 5**. For ancestral area reconstructions (AAR), the first two areas with the highest marginal probability (MP; in percent) are reported for both the parent and daughter nodes of each dispersal event. Age estimates are derived from Bayesian relaxed clock analysis treating priors on fossils as being drawn from a lognormal distribution. The mean date in million years ago and the 95% highest posterior density intervals are given for each parent and daughter node.*

## Discussion

### Divergence Ages Within Elaeagnaceae

Our results provided the first estimates for the stem and crown nodes of Elaeagnaceae (mean ages were estimated as 89.3 and 40.6 Ma, respectively). We note that the oldest known fossil of Elaeagnaceae (pollen of *Elaeagnacites* sp.) is from the Taizhou Formation in North Jiangsu Basin (Song and Qian, 1989), which in China corresponds to the Maastrichtian (Song et al., 2004). Our age estimate for this family thus predates its earliest known fossil record by some 20–30 Myr, indicating a considerable gap in the fossil record, similar to many other families of angiosperms (Crepet et al., 2004).

### Phylogenetic Relationships Among Species of *Hippophae*

We reconstructed the phylogenetic relationships of *Hippophae* based on thorough sampling across lineages/distribution ranges and a relatively large amount of sequence data of both cpDNA and nuclear DNA. The genus was strongly supported as monophyletic and can be divided into two distinct sub-clades: one comprising *H. rhamnoides* and *H. tibetana* and the other comprising *H*. *gyantsensis, H. neurocarpa*, and *H. salicifolia* (**Figures 2, 3**). The relationship between *H. rhamnoides* and *H. tibetana* was uncertain in earlier studies (Bartish et al., 2002; Sun et al., 2002; Jia et al., 2011). However, it received a strong Bayesian (PP = 1.00) and moderate ML bootstrap (BP > 84%) support in both phylogenies. The relationships among the three species within the other sub-clade are still poorly resolved in the cpDNA phylogeny and incongruent between the two data sets. This could probably result from the homoploid hybridizations between species falling into the two sub-clades, i.e., *H. neurocarpa* and *H. rhamnoides*, as indicated by our previous work based on population genetic data and niche modeling (Jia et al., 2016).

### Biogeographical Patterns in *Hippophae*

#### Early Diversification in *Hippophae* (Stage I)

The south QTP (the central Himalaya and southern Tibet) was suggested as the ancestral area of the genus in our reconstructions (**Figures 4, 5**). Early diversification within the genus most likely started in this region in the late Oligocene/early Miocene. This placement in time and space corresponds to the uplift-stage C of the QTP occurring at 25–20 Ma (Zhang et al., 2013). Reports from dated molecular phylogenies of plant taxa indicated additional cases of initial diversification in the south or east QTP at this period or soon after in *Androsace* (Roquet et al., 2013) and *Lepisorus* (Wang et al., 2012). It is tempting to link the earliest diversification in *Hippophae* and the other genera mentioned above with the rise of the southern QTP in the early Miocene. Nonetheless, we note that reconstruction of ages and paleoaltitudes of the QTP uplifts is still a controversial area of research (Harris, 2006; Molnar et al., 2010). For example, the central QTP has been argued to be already near its present elevation at least 40 Ma (Rowley and Currie, 2006; DeCelles et al., 2007; Wang C. et al., 2008), at least 15 Ma (Spicer et al., 2003), or no older than 2–3 Ma (Wang Y. et al., 2008). Only when this controversy has been resolved and robust models for early orogeneses of the QTP have been developed, can linking geological and biogeographical events around this plateau in the Paleogene and early Neogene be meaningful.

#### Diversification and Expansion of *H. rhamnoides* (Stage II)

After a period of initial diversification in the central Himalaya/southern QTP in the early to middle Miocene and differentiation of all species, diversification in the genus likely shifted to the eastern QTP (**Figures 4, 5**). This area was inferred as the ancestral area of *H. rhamnoides* and several oldest lineages of this species are distributed there. As indicated by our RASP analysis (**Figures 4, 5**), differentiation of a common ancestor of ssp. *sinensis* and *turkestanica* was followed by a dispersal event across the QTP in the earliest late Miocene (the Tortonian age) and an extinction event in the ancestral area of ssp. *turkestanica*. These events might be associated with uplift of the western QTP (Zhang et al., 2013) and concomitant paleoclimatic changes in the area, such as advance of more open and arid environments across the QTP (Tang and Shen, 1996; Hui et al., 2011).

Unlike in the early Neogene, links among paleoecology, climatic shifts, and orogeneses in the late Neogene (the last 10 Ma) are relatively well-studied. Considerable climatic and landscape modifications in area between the west QTP and Europe in the late Miocene have been indicated by geological (Ramstein et al., 1997; Trifonov et al., 2012), paleozoological (Koufos et al., 2005; Fortelius et al., 2006; Eronen et al., 2009), and paleobotanical (Mosbrugger et al., 2005; Kovar-Eder et al., 2008) data. These processes were coincident with ecosystem turnovers of this age in the area: degradation of tropical and subtropical laurophyllous forests, expansion of warm temperate sclerophyllous woodlands, scrubs, and savannas (Mosbrugger et al., 2005; Kovar-Eder et al., 2008; Biltekin, 2010), and expansion of grasses (Strömberg et al., 2007). Reconstructions of evolution of bovid and *Hipparion* megafauna (Barry et al., 1995; Fortelius et al., 2006; Eronen et al., 2009; Liu L. et al., 2012) and contemporary turnovers in small mammalian fauna (Mein, 2003; Maridet et al., 2007) across central parts of Eurasia provided a detailed evidence for these ecological and evolutionary processes in the late Miocene. Our results indicated that *H. rhamnoides* was involved in these processes. After dispersal to the west QTP, this species expanded across Karakoram, Hindu Kush, Pamir, and Tianshan, giving rise to extant ssp. *turkestanica*. Orogeneses during that geological epoch resulted in relatively close positioning of the western ranges of Hindu Kush, Kopet Dag, Elburz, and Caucasus (Trifonov et al., 2012). Their orientation created more or less a continuous belt of mountain ranges, which thus served as an ecological bridge between highland habitats in Central Asia, Anatolia, and Balkans. After differentiation within the ancestral area of the clade of Asia Minor/European subspecies in the west QTP, one or two of the diverged lineages could use this ecological opportunity for westward expansion. Our age estimates of the putative dispersals followed by local extinctions corresponded to a period of considerable orogenic, climatic, and ecological modifications in the Central Asia, eastern Anatolia, and eastern Europe (Mosbrugger et al., 2005; Fortelius et al., 2006; Trifonov et al., 2012). Taken together, our results implicated that *H. rhamnoides* originated in the middle Miocene in the east QTP and reached the west Europe in three long-distance dispersals in the late Miocene (**Figures 4, 5**). The main trigger of these dispersals seems to be arising of a relatively continuous belt of mountain ranges west of the QTP and opening of landscape through deforestation of these areas. Ecological and physiological characteristics of the species (see Introduction) allow it an efficient colonization of mountain landscapes and survival in these unstable ecosystems. Although *H. rhamnoides* is easily outcompeted from stable lowland forested ecosystems, this species can become dominant in ruderal and mostly arid ecosystems of mountain slopes and valleys (Igor V. Bartish, personal observations during field trips across Caucasus, Central Asia, Himalayas, and southern Siberia in 2012–2014).

Apart from *Hippophae*, other plants with ecological niches in open landscapes and relatively dry and cold climates have also been shown to disperse westwards out of the QTP at this period by molecular phylogenetic analyses, e.g., *Lilium* (Gao et al., 2013) and *Saussurea* (Wang Y.J. et al., 2009). We suggest additional molecular phylogenetic studies focusing on clades with Eurasian ranges and putative ancestral areas in the QTP and using dating and dispersal-vicariance analyses should be carried out to test the generality of this migration route in the late Miocene. For example, one can test the hypothesis of Bobrov (1962) that ancestors of *M. germanica* (L.) Desv., which currently shares with *H. rhamnoides* similar ranges and ecological niches, could have migrated to Europe from Central Asia taking the same route in the same geological epoch. On the other hand, testing directions and comparing ages of migrations across Eurasia of representatives of different ecological groups can lead to deeper insights into mechanisms of biotic turnovers on the continent.

#### Diversification and Dispersals Within Taxa (Stage III)

Our dating analyses revealed an interesting pattern: regardless of the age of each taxon, differentiation within all taxa (except the paraphyletic ssp. *yunnanensis*) was largely temporally coincident and relatively recent, in the late Pliocene/Pleistocene (**Figures 4, 5** and **Supplementary Tables S8, S9**). Specifically, results of our dating and ancestral area reconstructions from cpDNA (**Figure 4**) suggested two dispersal/vicariance events within taxa in the Pliocene: from the southern to north-eastern QTP in *H. tibetana* (dispersal 9) and from the eastern QTP to the Mongolian Plateau in *H. rhamnoides* (ssp. *sinensis/yunnanensis* clade and ssp. *mongolica*; dispersal 7). We note that both dispersals were in a northward direction. Three more northward dispersals from the eastern QTP, followed by vicariance, were inferred lately in the Pleistocene: one to northern China (ssp. *sinensis*, dispersal 8), and the other two to the northern QTP (*H. neurocarpa* ssp. *neurocarpa* and *stellatopilosa*; dispersals 12 and 13, respectively). Finally, our results suggested two almost coincident westward dispersals in the late Pliocene/early Pleistocene along opposite slopes of the Himalayas: northern slopes in *H. tibetana* (dispersal 10) and southern slopes in *H. salicifolia* (dispersal 11). The pattern of coincident biogeographical events in *Hippophae* in the Pliocene and Pleistocene with similar directions of dispersals (mostly from south to north) within different regions of the QTP is conspicuous. Noteworthy, this pattern was also found in European subspecies of *H. rhamnoides* in the Late Quaternary (Bartish et al., 2006). A growing number of molecular studies suggest diversifications within species and dispersals across or around the QTP at this period (for recent reviews, see Qiu et al., 2011; Liu J.Q. et al., 2012). Results of our study, together with the earlier reports, indicate a strong impact of the last stage of the QTP uplift since 5 Ma (Zhang et al., 2013) and concomitant climatic fluctuations on evolutionary processes in this hot spot of biodiversity (Myers et al., 2000; Mittermeier et al., 2005). It is likely that species responded to geological and climatic changes around the QTP by range expansions and fragmentations, long-distance dispersals, and local extinctions. General trends of these responses within different ecological groups of plants are still poorly known, but they likely depend on ecological characteristics of particular lineages. Spatial and temporal resolution of analyses on taxa with their highest diversity in the QTP area are increasing fast. These efforts will undoubtedly provide new insights into mechanisms of evolutionary responses of species with different ecological characteristics to climatic and geological processes in the late Neogene/Quaternary of Eurasia.

## Conclusion

Taxa with Eurasian ranges, centers of diversity around the QTP and different ecological characteristics can represent useful model systems to study the mechanisms of biotic responses to climatic fluctuations and orogeneses on the continent. However, few of these taxa have been included into comprehensive biogeographical analyses using ancestral area reconstructions and molecular dating, preventing development of a general understanding of the detailed mechanisms. In present study, we integrated phylogenetic, molecular dating, and biogeographical methods to reconstruct the evolutionary history of *Hippophae*, a pioneer plant with an extremely wide range of climatic niches in Eurasia and the highest intra-generic diversity around the QTP. Our results supported an old biogeographical hypothesis and suggested that multiple dispersals should have contributed to the observed biogeographical patterns of *Hippophae*. These dispersals can be grouped into three stages: (i) dispersals from ancestral area of the genus in the south QTP to the east QTP in the early Miocene; (ii) long-distance dispersals from the east QTP to Europe along rising mountain systems across Eurasia in the late Miocene; and (iii) mostly intraspecific northward and westward dispersals around the QTP and other mountain ranges in the Pliocene/Pleistocene. Additionally, at least three cases of possible extinctions were inferred in different parts of the QTP. We argue that biotic responses to environmental changes in the Neogene/Quaternary of Eurasia can depend on ecological characteristics of evolutionary lineages and result in different biogeographical patterns. To advance our understanding of mechanisms of association between paleoclimatic and macro-evolutionary processes, further studies should focus on comparison of evolutionary histories of lineages from ecologically and geographically similar groups.

## Author Contributions

D-RJ and IB conceived the ideas. D-RJ collected and analyzed the data. D-RJ and IB wrote the paper. All authors approved the final manuscript.

## Conflict of Interest Statement

The authors declare that the research was conducted in the absence of any commercial or financial relationships that could be construed as a potential conflict of interest.

## Footnotes

1 http://www.cvh.ac.cn

2 http://www.gbif.org
