# Supplementary material for: Climatic Changes and Orogeneses in the Late Miocene of Eurasia: The Main Triggers of an Expansion at a Continental Scale?
Source: Front Plant Sci. 2018 Sep 25;9:1400. doi: 10.3389/fpls.2018.01400 (PMC6167526; doi:10.3389/fpls.2018.01400)
Supplement: Supplementary file 1 [file Data_Sheet_1.pdf]

Supplementary Material

Climatic Changes and Orogeneses in the Late Miocene of Eurasia: the Main Triggers of an Expansion at a Continental Scale?

Dong-Rui Jia[1, 2, 3*] and Igor V. Bartish[3*]

[1]*School of Ecology and Environmental Science & Yunnan Key Laboratory for Plateau Mountain Ecology and Restoration of Degraded Environments, Yunnan University, Kunming, China*

[2]*Department of Botany, Faculty of Science, Charles University in Prague, Prague, Czech Republic*

[3]*Department of Genetic Ecology, Institute of Botany, Academy of Sciences of the Czech Republic, Pruhonice, Czech Republic*

***Correspondence:**

Dong-Rui Jia, jia.drui@ynu.edu.cn; Igor V. Bartish, igor.bartish@ibot.cas.cz

1 Supplementary Tables

Table S1 Taxa and GenBank accession numbers for sequences used in reconstructing the phylogeny of Rosales.

Family	Genus	18S	26S	*atp*B	*mat*K	*ndh*F	*psb*BTNH	*rbc*L	*rpo*C2	*rps*4	*trn*L-F
**Rosales**											
Barbeyaceae											
	Barbeya	JF317358	JF317379	JF317398	JF317418	JF317457	JF317477	JF317437	JF317497	JF317517	AJ225795
Cannabaceae											
	Cannabis	JF317360	EU002151	JF317400	AF345317	AY289250	EU002390	AJ402933	EU002481	EU002301	AF501598
	Celtis	JF317361	JF317380	JF317401	JF317420	JF317459	JF317479	JF317439	JF317499	JF317519	AY488673
	Trema	JF317376	JF317395	JF317415	JF317434	JF317474	JF317494	JF317454	JF317514	JF317532	JN040388
Dirachmaceae											
	Dirachma	JF317364	JF317383	–	JF317423	JF317462	JF317482	JF317442	JF317502	JF317522	AJ225796
Elaeagnaceae											
	Elaeagnus[1]	JF317366	JF317385	JF317405	JF317425	JF317464	JF317484	JF317444	JF317504	JF317524	–
	Elaeagnus[2]	–	–	–	–	–	–	U17038	–	–	DQ838727
	Elaeagnus[3]	L24090	–	–	AY257529	–	–	HM849968	–	–	JN102138
	Hippophae[4]	JF317370	JF317389	JF317409	JF317428	JF317468	JF317488	JF317448	JF317508	JF317526	JQ289181
	Hippophae[5]	–	–	–	JF954063	–	–	JF941954	–	–	HM769675
	Hippophae[6]	–	–	–	JF954058	–	–	U59821	–	–	HM769681
	Shepherdia[7]	–	–	–	–	–	–	AJ225787	–	–	AJ225794
	Shepherdia[8]	AF207020	AF479100	AF209675	JN966666	–	–	U17039	–	–	GQ245525
Moraceae											
	Broussonetia	JF317359	AY686805	JF317399	JF317419	JF317458	JF317478	JF317438	JF317498	JF317518	AF501602
	Cudrania	JF317362	JF317381	JF317402	JF317421	JF317460	JF317480	JF317440	JF317500	JF317520	JN102171
	Ficus	JF317367	JF317386	JF317406	JF317426	JF317465	JF317485	JF317445	JF317505	JF317525	JN040373
	Morus	L24398	AF479232	NC008359	NC008359	NC008359	NC008359	NC008359	NC008359	NC008359	AF501608

Table S1 *(continued)*

Family	Genus	18S	26S	*atp*B	*mat*K	*ndh*F	*psb*BTNH	*rbc*L	*rpo*C2	*rps*4	*trn*L-F
Rhamnaceae											
	Ceanothus	U42799	AF479102	AF209558	AF049815	EU002210	EU002393	U06795	EU002484	EU002304	HQ325601
	Gouania	JF317369	JF317388	JF317408	JF317427	JF317467	JF317487	JF317447	JF317507	–	AJ390344
	Hovenia	JF317371	JF317390	JF317410	JF317429	JF317469	JF317489	JF317449	JF317509	JF317527	AJ390343
	Rhamnus	JF317374	JF317393	JF317413	JF317432	JF317472	JF317492	JF317452	JF317512	JF317530	AF348565
Rosaceae											
	Dryas	JF317365	JF317384	JF317404	JF317424	JF317463	JF317483	JF317443	JF317503	JF317523	DQ851231
	Filipendula	JF317368	JF317387	JF317407	AB073684	JF317466	JF317486	JF317446	JF317506	–	AJ416463
	Neillia	JF317372	JF317391	JF317411	JF317430	JF317470	JF317490	JF317450	JF317510	JF317528	AF487228
	Sanguisorba	JF317375	JF317394	JF317414	JF317433	JF317473	JF317493	JF317453	JF317513	JF317531	AY634775
	Spiraea	U42801	AF479103	AJ235608	AF288127	EU002259	EU002451	L11206	EU002541	EU002362	AF348571
Ulmaceae											
	Ulmus	JF317377	JF317396	JF317416	JF317435	JF317475	JF317495	JF317455	JF317515	JF317533	AF501593
	Zelkova	U42819	AF479099	AF209699	AF345328	EU002273	EU002465	AF206835	EU002556	–	JX399109
Urticaceae											
	Debregeasia	JF317363	JF317382	JF317403	JF317422	JF317461	JF317481	JF317441	JF317501	JF317521	JN040372
	Pilea	JF317373	JF317392	JF317412	JF317431	JF317471	JF317491	JF317451	JF317511	JF317529	DQ179359
	Boehmeria	JF317378	JF317397	JF317417	JF317436	JF317476	JF317496	JF317456	JF317516	JF317534	AY208722
**Cucurbitales(Outgroup)**											
Begoniaceae											
	Begonia	AF008950	AY968403	AY968426	AY968445	EU002200	EU002384	L01888	EU002475	EU002294	GU397135

Note: [1]*Elaeagnus bockii*; [2]*Elaeagnus angustifolia*; [3]*Elaeagnus umbellata*; [4]*Hippophae rhamnoides* subsp. *sinensis*; [5]*Hippophae tibetana*; [6]*Hippophae salicifolia*; [7]*Shepherdia argentea* and [8]*Shepherdia canadensis*.

Table S2 Sequences of primers used for amplification and sequencing of DNA fragments from plastid and nuclear genomes of *Hippophae* L.

Locus	Primer sequence 5'–3'		Reference
*trn*C^{GCA}-ycf6	F:	CCAGTTCRAATCYGGGTG	(Shaw et al., 2005)
	R:	GCCCAAGCRAGACTTACTATATCCAT	
*trn*D^{GUC}-*trn*T^{GGU}	F:	ACCAATTGAACTACAATCCC	(Demesure et al., 1995)
	R:	CTACCACTGAGTTAAAAGGG	
*trn*L^{UAA}-*trn*F^{GAA}	F:	CGAAATCGGTAGACGCTACG	(Taberlet et al., 1991)
	R:	ATTTGAACTGGTGACACGAG	
*trn*S^{UGA}-*trn*fM^{CAU}	F:	GAGAGAGAGGGATTCGAACC	(Demesure et al., 1995)
	R:	CATAACCTTGAGGTCACGGG	
*trn*S^{GCU}-*trn*G^{UCC}	F:	GCCGCTTTAGTCCACTCAGC	(Hamilton, 1999)
	R:	GAACGAATCACACTTTTACCAC	
*At*103	F:	CTTCAAGCCMAAGTTCATCTTCTA	(Li et al., 2008)
	R:	TTGGCAATCATTGAGGTACATNGTMACATA	
G3pdh	F:	GATAGATTTGGAATTGTTGAGG	(Strand et al., 1997)
	R:	AAGCAATTCCAGCCTTGG	
ITS	F:	AGAAGTCGTAACAAGGTTTCCGTAGG	(White et al., 1990)
	R:	TCCTCCGCTTATTGATATGC	
Ms	F:	GGAAGATGRTCATCAAYGCNCTYAAYTC	(Lewis and Doyle, 2001)
	R:	GTCTTNACRTAGCTGAADATRTARTCCC	
Tpi	F:	AAGGTCATTGCATGTGTTGG	(Strand et al., 1997)
	R:	CTTTACCAGTTCCAATAGCCC	

Table S3 Taxa, population codes and GenBank accession numbers for sequences used in analysis of *Hippophae* L.

Taxon	Population	*trn*C-ycf6	*trn*D-T	*trn*L-F	*trn*S-fM	*trn*S-G	*At*103	*G3pdh*	ITS	*Ms*	*Tpi*
H. salicifolia	4079	KF620857	KF620916	KF620652	KF620729	KF620763	KF620838	KF620955	KF620619	KF620691	KF620799
	09XZ040	KF620858	KF620917	KF620653	KF620730	KF620764	KF620839	KF620956	KF620620	KF620692	KF620800
	P	KF620856	KF620918	KF620654	KF620731	KF620765	KF620840	KF620957	KF620621	–	KF620801
H. gyantsensis	06252	KF620847	KF620886	KF620627	KF620699	KF620738	KF620808	KF620925	KF620594	KF620661	KF620769
	06217	KF620848	KF620887	KF620628	KF620700	KF620739	KF620809	KF620926	KF620595	KF620662	KF620770
	2568	KF620849	KF620888	KF620629	KF620701	KF620740	KF620810	KF620927	KF620596	KF620663	KF620771
H. neurocarpa											
ssp. *neurocarpa*	YNG1	KF620850	KF620889	KF620630	KF620702	KF620741	KF620811	KF620928	KF620597	KF620664	KF620772
	1522	KF620851	KF620890	KF620631	KF620703	KF620742	KF620812	KF620929	KF620598	KF620665	KF620773
	MM-31	KF620852	KF620891	KF620632	KF620704	KF620743	KF620813	KF620930	KF620599	KF620666	KF620774
ssp. *stellatopilosa*	Ao129	KF620853	KF620892	KF620633	KF620705	KF620744	KF620814	KF620931	KF620600	KF620667	KF620775
	QML1	KF620854	KF620893	KF620634	KF620706	KF620745	KF620815	KF620932	KF620601	KF620668	KF620776
	Ao111	KF620855	KF620894	KF620635	KF620707	KF620746	KF620816	KF620933	KF620602	KF620669	KF620777
H. tibetana	07138	KF620881	KF620919	KF620655	KF620732	KF620766	KF620841	KF620958	KF620622	KF620693	KF620802
	Henan	KF620882	KF620920	KF620656	KF620734	KF620767	KF620842	KF620959	KF620623	KF620694	KF620803
	ST	KF620880	KF620921	KF620657	–	KF620768	KF620843	KF620960	KF620624	KF620695	KF620804
H. rhamnoides											
ssp. *yunnanensis*	06309	KF620877	KF620913	KF620649	KF620726	KF620755	KF620835	KF620952	KF620616	KF620688	KF620796
	06321	KF620878	KF620914	KF620650	KF620727	KF620761	KF620836	KF620953	KF620617	KF620689	KF620797
	06324	KF620879	KF620915	KF620651	KF620728	KF620762	KF620837	KF620954	KF620618	KF620690	KF620798
ssp. *sinensis*	Ao96	KF620871	KF620907	KF620643	KF620720	KF620758	KF620829	KF620946	KF620610	KF620682	KF620790
	05276	KF620872	KF620908	KF620644	KF620721	KF620759	KF620830	KF620947	KF620611	KF620683	KF620791
	05179	KF620873	KF620909	KF620645	KF620722	KF620760	KF620831	KF620948	KF620612	KF620684	KF620792

Table S3 *(continued)*

Taxon	Pop. code	*trn*C-ycf6	*trn*D-T	*trn*L-F	*trn*S-fM	*trn*S-G	*At*103	*G3pdh*	ITS	*Ms*	*Tpi*
H. rhamnoides											
ssp. *turkestanica*	GL	KF620875	KF620910	KF620646	KF620723	KF620751	KF620832	KF620949	KF620613	KF620685	KF620793
	SKZ	KF620874	KF620911	KF620647	KF620724	KF620752	KF620833	KF620950	KF620614	KF620686	KF620794
	12413	KF620876	KF620912	KF620648	KF620725	KF620753	KF620834	KF620951	KF620615	KF620687	KF620795
ssp. *mongolica*	05047	KF620869	KF620903	JQ289182	KF620716	JQ289198	KF620825	KF620942	JQ289227	KF620678	KF620786
	6667	KF620867	KF620904	KF620640	KF620717	KF620757	KF620826	KF620943	KF620607	KF620679	KF620787
	6657	KF620868	KF620905	KF620641	KF620718	KF620756	KF620827	KF620944	KF620608	KF620680	KF620788
ssp. *caucasica*	0402	KF620861	KF620897	JQ663586	KF620710	JQ663592	KF620819	KF620936	JQ663578	KF620672	KF620780
	21286	KF620862	KF620898	KF620636	KF620711	KF620747	KF620820	KF620937	KF620603	KF620673	KF620781
	9835	KF620863	KF620899	KF620637	KF620712	KF620748	KF620821	KF620938	KF620604	KF620674	KF620782
ssp. *fluviatilis*	SW1	KF620866	KF620900	JQ289193	KF620713	JQ289217	KF620822	KF620939	JQ289287	KF620675	KF620783
	6757	KF620864	KF620901	KF620638	KF620714	KF620749	KF620823	KF620940	KF620605	KF620676	KF620784
	6682	KF620865	KF620902	KF620639	KF620715	KF620750	KF620824	KF620941	KF620606	KF620677	KF620785
ssp. *carpatica*	9897	KF620859	KF620895	JQ663584	KF620708	JQ663591	KF620817	KF620934	JQ663577	KF620670	KF620778
	9898	KF620860	KF620896	JQ663585	KF620709	JQ663595	KF620818	KF620935	JQ663576	KF620671	KF620779
ssp. *rhamnoides*	6672	KF620870	KF620906	KF620642	KF620719	KF620754	KF620828	KF620945	KF620609	KF620681	KF620789
Elaeagnus triflora	IVB-29	KF620844	KF620883	KF620625	KF620696	KF620735	KF620805	KF620922	KF620592	KF620658	–
Elaeagnus umbellata	07078	KF620845	KF620884	HM769678	KF620697	KF620736	KF620806	KF620923	HM769715	KF620659	–
Shepherdia argentea	6777	KF620846	KF620885	KF620626	KF620698	KF620737	KF620807	KF620924	KF620593	KF620660	–
Rhamnus davurica		–	–	AY626420	–	–	–	–	AY626441	–	–

Table S4 Fossils used for calibration of phylogenetic trees of Rosales and *Hippophae* in dating analyses (see Supplementary Figure S2 and Figure 2). Absolute ages in Ma used for calibration were determined as the minimum age of each geological stratum relying on the Geological Time Scale v.4.0 (Walker et al., 2012). Assignments of fossils to extant taxa are [a)] "intuitive" or [b)]"apomorphy-based", as specified by Sauquet *et al.* (2012). [c)] The fossil record was used to calibrate the stem node of a monophyletic group of four western subspecies within *H. rhamnoides* (Jia et al., 2012).

Fossil	Taxon name	Family	Fossil organ	Locality	Region	Geological age	Absolute age (Ma)
1	*Triorites minutipori* Muller	Ulmaceae[a)]	pollen	Sarawak	South-eastern Asia	Turonian	89.8
2	*Celtis aspera* Manchester, Akhmetiev & Kodrul	Cannabaceae[b)]	fruits, leaves	Numerous localities	North America; Asia	Paleocene	56
3	*Ficus* L.	Moraceae[a)]	fruit	Lower Bagshot	Dorset, England	Ypresian	47.8
4	*Paliurus clarnensis* Burge & Manchester	Rhamnaceae[b)]	fruit	Red Gap, Jefferson	Oregon, USA	Lutetian	41.2
5	*Shepherdia weaveri* Becker	Elaeagnaceae[a)]	leaves	Mormon Creek	Montana, USA	Priabonian	33.9
6	*Hippophae rhamnoides* L.	Elaeagnaceae[c)]	pollen	Burhanli	Marmor sea, Turkey	Tortonian	7.2

Table S5 Gene/partition characteristics of the Rosales dataset.

Partition	No. of taxa	Aligned length	Variable sites	Parsimony-informative sites	Model selected by AIC
18S rDNA	28	1733	202	101	GTR + I + G
26S rDNA	27	3256	494	277	GTR + I + G
*atp*B	26	1242	286	169	TIM1 + I + G
*mat*K	30	1029	535	333	TVM + I + G
*ndh*F	26	2116	924	592	GTR + I + G
*psb*BTNH	26	2278	598	354	TVM + I + G
*rbc*L	32	1289	315	195	GTR + I + G
*rpo*C2	26	3876	1547	908	GTR + I + G
*rps*4	23	546	164	79	GTR + G
*trn*L-F	31	970	502	311	GTR + I + G
Total	32	18335	5567	3319	

Table S6 Gene/partition characteristics of the *Hippophae* L. datasets.

Data set	Partition	No. of Taxa	Aligned lengths	Variable sites	Parsimony-informative sites	Model selected by AIC
cpDNA						
	*trn*C-ycf6	39	928	129	96	TPM1uf + G
	*trn*D-T	39	1396	144	99	TPM1uf + I + G
	*trn*L-F	40	959	198	54	TIM1 + G
	*trn*S-fM	38	1441	187	133	TIM1 + G
	*trn*S-G	39	738	88	60	TrN + G
	Total	40	5462	746	442	
nuclear DNA						
	*At*103	39	524	65	46	HKY + I
	G3pdh	39	735	42	29	HKY + I
	ITS	40	714	276	174	GTR + I
	Ms	38	610	69	53	TPM1uf + G
	Tpi	36	375	40	22	TPM3uf + I
	Total	40	2958	492	324	

Table S7 Divergence time estimates of diversifications among and within families of Rosales. Ranges on PL divergence time represent ± 1 standard deviation of the mean based on 1000 bootstrap replicates; while ranges on Bayesian estimates represent 95% confidence limits around mean age estimates. All ages are millions of years.

Node	Age					
	PL		UCLN-lognormal		UCLN-uniform	
Rosales$_{crown}$	103	(102.84–103.15)	102.53	(101.66–103.00)	102.56	(101.69–103.00)
Rosaceae$_{crown}$	75.93	(72.71–77.63)	69.26	(61.43–77.18)	69.83	(61.50–77.78)
Subclades split	96.22	(95.60–96.89)	95.96	(94.22–97.73)	96.13	(94.33–97.93)
Ulmaceae$_{stem}$	89.80	(89.58–90.27)	90.45	(89.84–91.40)	90.64	(89.80–92.03)
Ulmaceae$_{crown}$	7.34	(5.69–9.23)	6.92	(4.53–9.46)	6.89	(4.67–9.46)
Canabaceae$_{stem}$	76.26	(75.38–77.32)	76.40	(73.30–79.48)	76.43	(72.62–79.74)
Canabaceae$_{crown}$	58.52	(57.04–60.36)	57.11	(56.04–59.10)	58.47	(56.62–62.35)
Moraceae$_{stem}$	73.69	(72.76–74.74)	73.53	(70.12–76.92)	73.56	(69.97–77.15)
Urticaceae$_{crown}$	56.55	(55.13–58.24)	55.42	(49.90–60.82)	55.55	(49.82–60.87)
Moraceae$_{crown}$	55.71	(54.77–56.62)	56.14	(53.53–58.97)	56.32	(53.43–59.05)
Rhamnacea$_{stem}$	93.22	(92.52–94.18)	91.09	(87.52–93.98)	91.34	(88.33–94.16)
Rhamnacea$_{crown}$	77.52	(75.72–79.01)	76.15	(70.07–82.42)	76.46	(70.49–82.32)
Elaeagnaceae$_{stem}$	92.25	(91.13–93.06)	89.25	(85.72–92.97)	89.52	(86.26–92.94)
Barbeyaceae$_{stem}$	86.61	(84.14–88.07)	82.81	(77.16–87.88)	83.16	(77.75–88.14)
Elaeagnaceae$_{crown}$	42.19	(40.94–43.79)	40.62	(36.94–44.04)	40.67	(37.61–44.12)
Elaeagnus$_{crown}$	20.54	(18.13–23.98)	15.68	(8.85–23.47)	15.51	(8.75–23.04)
Shepherdia$_{stem}$	33.90	(33.27–34.57)	34.38	(33.93–35.07)	34.40	(33.90–35.43)
Shepherdia$_{crown}$	19.76	(16.11–24.43)	12.31	(2.73–29.51)	10.89	(2.69–28.05)
Hippophae$_{crown}$	19.53	(16.51–23.15)	8.30	(4.24–12.76)	8.21	(4.11–12.80)

Table S8 Ancestral area reconstructions (AAR) and divergence time estimates of *Hippophae* L. based on cpDNA data. Ancestral areas were constructed using Bayesian dispersal-vicariance (Bayes-DIVA) and dispersal-extinction-cladogenesis (DEC) models both implemented in RASP. The first two reconstructions with the highest probability are presented, and the marginal probability (MP; for Bayes-DIVA model) and relative probability (RP; for DEC model) for each reconstruction is given. Estimated ages are millions of years and represent results of three different analyses: Penalised Likelihood (PL), Bayesian UCLN-lognormal and Bayesian UCLN-uniform. Ranges on PL divergence time represent ± 1 standard deviation of the mean based on 1000 bootstrap replicates; while ranges on Bayesian estimates represent 95% confidence limits around mean age estimates. BEAST posterior probabilities (PP) are also provided for each node. Codes of areas follow Fig. 3.

Node	PP	Bayes-DIVA model				DEC model				Dating					
		1	MP	2	MP	1	RP	2	RP	PL		UCLN-lognormal		UCLN-uniform	
Hippophae$_{\text{stem}}$	1.00	A	68.82	B	17.61	A	0.26	B	0.15	33.90	(32.88–35.91)	34.88	(33.93–36.58)	35.94	(33.90–39.37)
Hippophae$_{\text{crown}}$	1.00	A	81. 07	B	11.19	AB	0.35	A	0.30	18.77	(17.72–22.82)	21.20	(18.30–24.00)	21.57	(18.38–25.00)
H. gyantsensis$_{\text{stem}}$	1.00	A	89.79	AB	5.64	A	0.70	AB	0.17	17.82	(15.09–22.99)	19.70	(16.73–22.63)	20.03	(16.69–23.44)
H. gyantsensis$_{\text{crown}}$	1.00	A	99.34	AD	0.29	A	1.00	–	–	1.93	(0.47–4.35)	2.46	(1.18–3.82)	2.51	(1.15–3.94)
H. salicifolia$_{\text{stem}}$	1.00	A	67.04	B	14.96	A	0.45	AB	0.22	16.85	(14.16–21.92)	17.57	(14.49–20.52)	17.89	(14.55–21.20)
H. salicifolia$_{\text{crown}}$	1.00	A	75.89	AD	10.95	AD	1.00	–	–	3.61	(2.41–5.51)	2.54	(1.24–4.01)	2.57	(1.20–4.10)
H. neurocarpa$_{\text{crown}}$	1.00	B	87.37	AB	4.32	BC	0.53	B	0.47	3.55	(2.51–5.49)	4.36	(2.86–5.88)	4.40	(2.93–6.05)
ssp. *stellatopilosa*$_{\text{crown}}$	0.94	B	95.00	BC	3.72	B	0.79	BC	0.21	3.07	(1.96–4.75)	3.62	(2.22–5.17)	3.66	(2.18–5.14)
ssp. *neurocarpa*$_{\text{crown}}$	1.00	B	79.00	BC	11.70	BC	1.00	–	–	0.34	(0.00–1.16)	0.71	(0.17–1.27)	0.73	(0.16–1.34)
H. tibetana$_{\text{stem}}$	1.00	A	52.74	B	31.81	AB	0.34	A	0.21	16.65	(13.43–22.51)	18.43	(15.72–21.46)	18.75	(15.66–22.01)
H. tibetana$_{\text{crown}}$	1.00	A	86.42	B	2.84	AD	0.54	AC	0.46	2.99	(1.62–5.23)	3.63	(2.15–5.31)	3.70	(2.12–5.32)
H. rhamnoides$_{\text{crown}}$	1.00	B	96.69	AB	2.64	B	0.64	AB	0.19	11.43	(9.93–14.76)	12.91	(10.72–15.19)	13.07	(10.69–15.62)
East/West QTP split	0.91	B	94.35	BD	1.36	B	0.44	BD	0.27	7.99	(7.02–9.67)	8.81	(7.82–9.96)	8.86	(7.76–10.16)
ssp. *mongolica*$_{\text{stem}}$	1.00	B	88.47	BF	6.66	BF	1.00	–	–	2.69	(1.63–4.53)	3.66	(2.36–5.14)	3.70	(2.27–5.14)

Table S8 *(continued)*

Node	PP	Bayes-DIVA model				DEC model				Dating					
		1	MP	2	MP	1	RP	2	RP	PL		UCLN-lognormal		UCLN-uniform	
ssp. *mongolica*$_{crown}$	1.00	F	93.28	BF	5.55	F	1.00	–	–	1.16	(0.49–2.11)	1.63	(0.77–2.60)	1.63	(0.75–2.54)
ssp. *sinensis*$_{crown}$	1.00	B	93.34	BE	4.66	BE	1.00	–	–	0.72	(0.00–1.55)	1.10	(0.34–2.03)	1.10	(0.30–2.01)
ssp. *turkestanica*$_{stem}$	1.00	D	53.60	B	27.29	B	0.16	D	0.14	7.20	(6.44–8.44)	7.65	(7.23–8.23)	7.66	(7.20–8.46)
ssp. *turkestanica*$_{crown}$	1.00	D	99.11	BD	0.32	D	0.87	BD	0.13	2.92	(2.08–4.25)	3.44	(2.09–4.82)	3.48	(2.18–4.91)
ssp. *caucasica*$_{stem}$	1.00	G	69.90	I	7.44	GH	0.16	GI	0.15	5.24	(4.54–6.51)	5.84	(4.67–7.11)	5.84	(4.49–7.09)
ssp. *caucasica*$_{crown}$	1.00	G	99.44	GH	0.12	G	1.00	–	–	1.77	(1.09–2.74)	2.07	(1.08–3.15)	2.10	(1.09–3.21)
ssp. *fluviatilis*$_{stem}$	1.00	H	44.15	I	37.41	HI	0.38	H	0.20	4.39	(3.50–5.43)	4.58	(3.20–6.02)	4.59	(3.20–6.11)
ssp. *fluviatilis*$_{crown}$	1.00	I	98.58	HI	1.04	I	1.00	–	–	0.53	(0.17–1.04)	0.91	(0.24–1.69)	0.90	(0.26–1.69)
ssp. *carpatica*$_{stem}$	1.00	H	98.89	HI	0.56	H	1.00	–	–	0.89	(0.28–1.43)	1.09	(0.35–1.95)	1.09	(0.35–1.95)
ssp. *carpatica*$_{crown}$	1.00	H	99.70	GH	0.08	H	1.00	–	–	0.03	(0.00–0.21)	0.20	(0.00–0.60)	0.21	(0.00–0.62)

Table S9 Ancestral area reconstructions (AAR) and divergence time estimates of *Hippophae* L. based on nDNA data. Ancestral areas were constructed using Bayesian dispersal-vicariance (Bayes-DIVA) and dispersal-extinction-cladogenesis (DEC) models both implemented in RASP. The first two reconstructions with the highest probability are presented, and the marginal probability (MP; for Bayes-DIVA model) and relative probability (RP; for DEC model) for each reconstruction is given. Estimated ages are millions of years and represent results of three different analyses: Penalised Likelihood (PL), Bayesian UCLN-lognormal and Bayesian UCLN-uniform. Ranges on PL divergence time represent ± 1 standard deviation of the mean based on 1000 bootstrap replicates; while ranges on Bayesian estimates represent 95% confidence limits around mean age estimates. BEAST posterior probabilities (PP) are also provided for each node. Codes of areas follow Fig. 3.

Node	PP	Bayes-DIVA model				DEC model				Dating					
		1	MP	2	MP	1	RP	2	RP	PL		UCLN-lognormal		UCLN-uniform	
Hippophae$_{stem}$	1.00	A	51.33	B	24.27	A	0.27	B	0.19	33.90	(33.01–35.30)	35.08	(33.94–37.21)	36.50	(33.90–39.77)
Hippophae$_{crown}$	1.00	A	58.67	B	27.13	AB	0.23	A	0.19	18.63	(16.37–22.72)	22.85	(17.10–28.41)	23.03	(17.29–29.23)
H. gyantsensis$_{stem}$	1.00	A	78.51	AB	7.57	AB	0.43	AC	0.35	7.57	(5.35–11.28)	11.55	(6.07–17.68)	11.32	(6.03–17.14)
H. gyantsensis$_{crown}$	1.00	A	99.32	*	0.68	A	1.00	–	–	2.08	(0.81–4.43)	3.77	(1.02–7.05)	3.95	(1.21–7.33)
H. salicifolia$_{stem}$	1.00	A	86.56	*	13.44	A	0.34	AB	0.24	9.77	(7.43–14.17)	15.56	(9.00–22.40)	15.12	(8.80–21.71)
H. salicifolia$_{crown}$	1.00	A	81.58	AD	9.07	AD	1.00	–	–	2.57	(0.14–6.58)	2.28	(0.18–5.51)	1.97	(0.14–4.66)
H. neurocarpa$_{crown}$	1.00	B	41.67	C	30.27	BC	0.77	B	0.12	1.00	(0.00–3.60)	2.93	(0.84–5.71)	2.65	(0.65–5.13)
ssp. *stellatopilosa*$_{crown}$	–	–	–	–	–	–	–	–	–	–	–	–	–	–	–
ssp. *neurocarpa*$_{crown}$	–	–	–	–	–	–	–	–	–	–	–	–	–	–	–
H. tibetana$_{stem}$	1.00	B	65.25	A	16.89	AB	0.22	A	0.14	16.93	(14.01–20.35)	18.86	(13.65–24.49)	19.48	(14.00–25.23)
H. tibetana$_{crown}$	1.00	C	53.04	A	20.82	AC	0.42	CD	0.32	5.06	(2.80–8.57)	6.61	(2.33–11.85)	6.40	(2.44–11.26)
H. rhamnoides$_{crown}$	1.00	B	98.05	*	1.95	B	0.65	BD	0.22	9.70	(7.74–12.75)	12.60	(9.45–16.39)	12.54	(9.02–16.27)
East/West QTP split	0.39	B	58.29	E	25.59	B	0.49	BD	0.28	8.50	(7.05–11.24)	10.15	(7.98–12.61)	10.16	(7.90–12.82)
ssp. *mongolica*$_{stem}$	1.00	D	95.39	*	4.61	DF	1.00	–	–	0.69	(0.00–1.76)	1.32	(0.19–2.78)	1.32	(0.23–2.72)

Table S9 *(continued)*

Node	PP	Bayes-DIVA model				DEC model				Dating					
		1	MP	2	MP	1	RP	2	RP	PL		UCLN-lognormal		UCLN-uniform	
ssp. *mongolica*$_{crown}$	1.00	F	93.52	*	5.45	F	1.00	–	–	0.03	(0.00–0.49)	0.43	(0.01–1.13)	0.42	(0.01–1.12)
ssp. *sinensis*$_{crown}$	1.00	E	84.52	BE	11.18	BE	1.00	–	–	0.51	(0.00–2.46)	1.05	(0.07–2.70)	0.95	(0.03–2.47)
ssp. *turkestanica+mongolica* $_{stem}$	1.00	D	61.11	B	9.68	BD	0.13	DI	0.12	7.20	(6.05–9.03)	7.86	(7.24–8.88)	8.00	(7.20–9.55)
ssp. *turkestanica*$_{crown}$	–	–	–	–	–	–	–	–	–	–	–	–	–	–	–
ssp. *caucasica*$_{stem}$	–	–	–	–	–	–	–	–	–	–	–	–	–	–	–
ssp. *caucasica*$_{crown}$	–	–	–	–	–	–	–	–	–	–	–	–	–	–	–
ssp. *fluviatilis*$_{stem}$	1.00	I	45.54	G	36.23	GI	0.40	HI	0.18	4.67	(3.38–6.28)	5.28	(3.08–7.63)	5.29	(2.85–7.62)
ssp. *fluviatilis*$_{crown}$	1.00	I	99.15	*	0.85	I	1.00	–	–	0.68	(0.00–1.90)	1.51	(0.22–3.24)	1.45	(0.23–3.07)
ssp. *carpatica*$_{stem}$	–	–	–	–	–	–	–	–	–	–	–	–	–	–	–
ssp. *carpatica*$_{crown}$	–	–	–	–	–	–	–	–	–	–	–	–	–	–	–

Asterisk (*) represent reconstructions with marginal probability values less than 5%.

2 Supplementary Figures

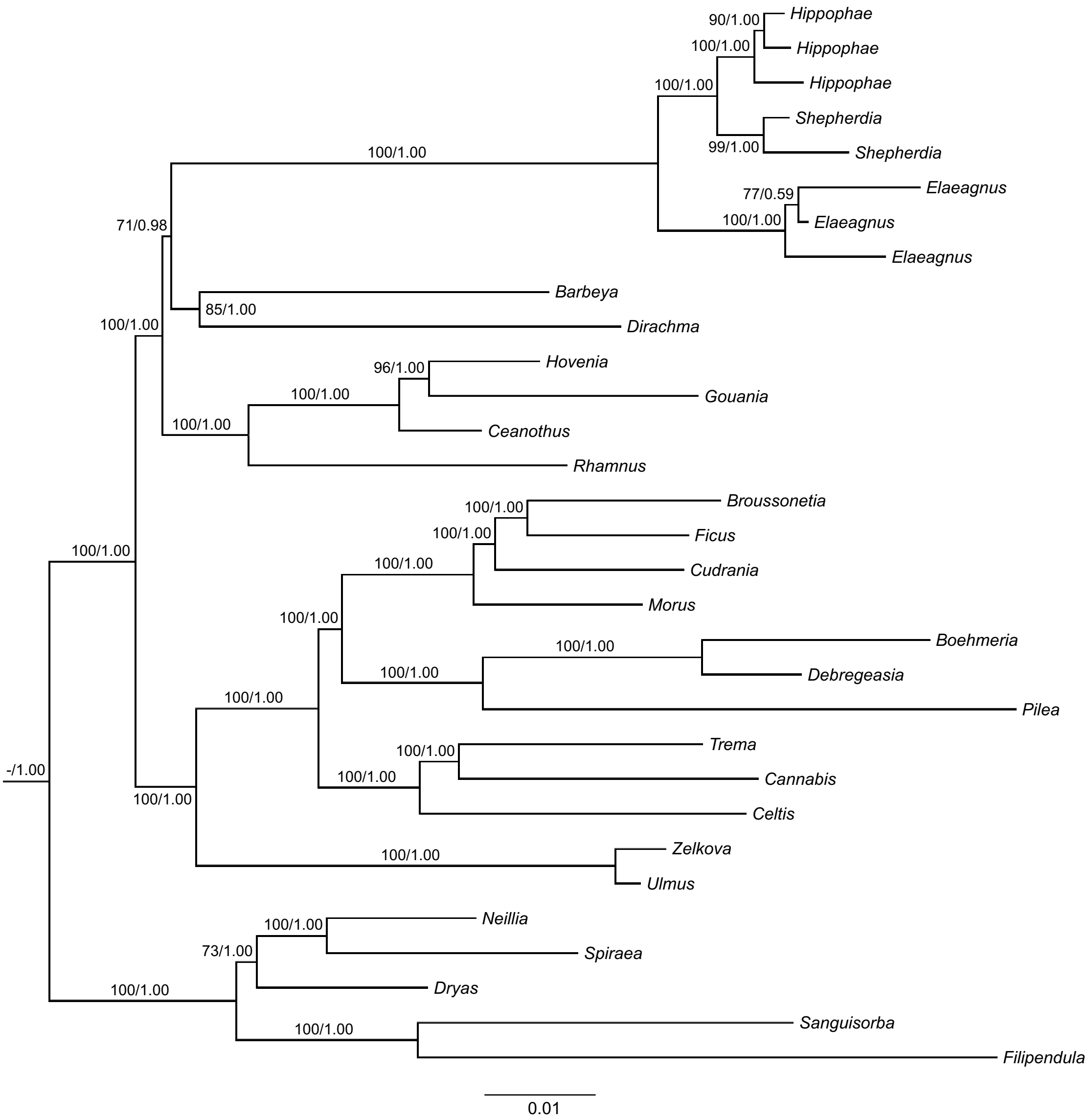

Figure S1. The ML tree of Rosales based on a data set consisting of 18335 aligned nucleotide sites for 13 gene partitions. Support values (ML Bootstrap/Bayesian posterior probability) are provided at nodes.

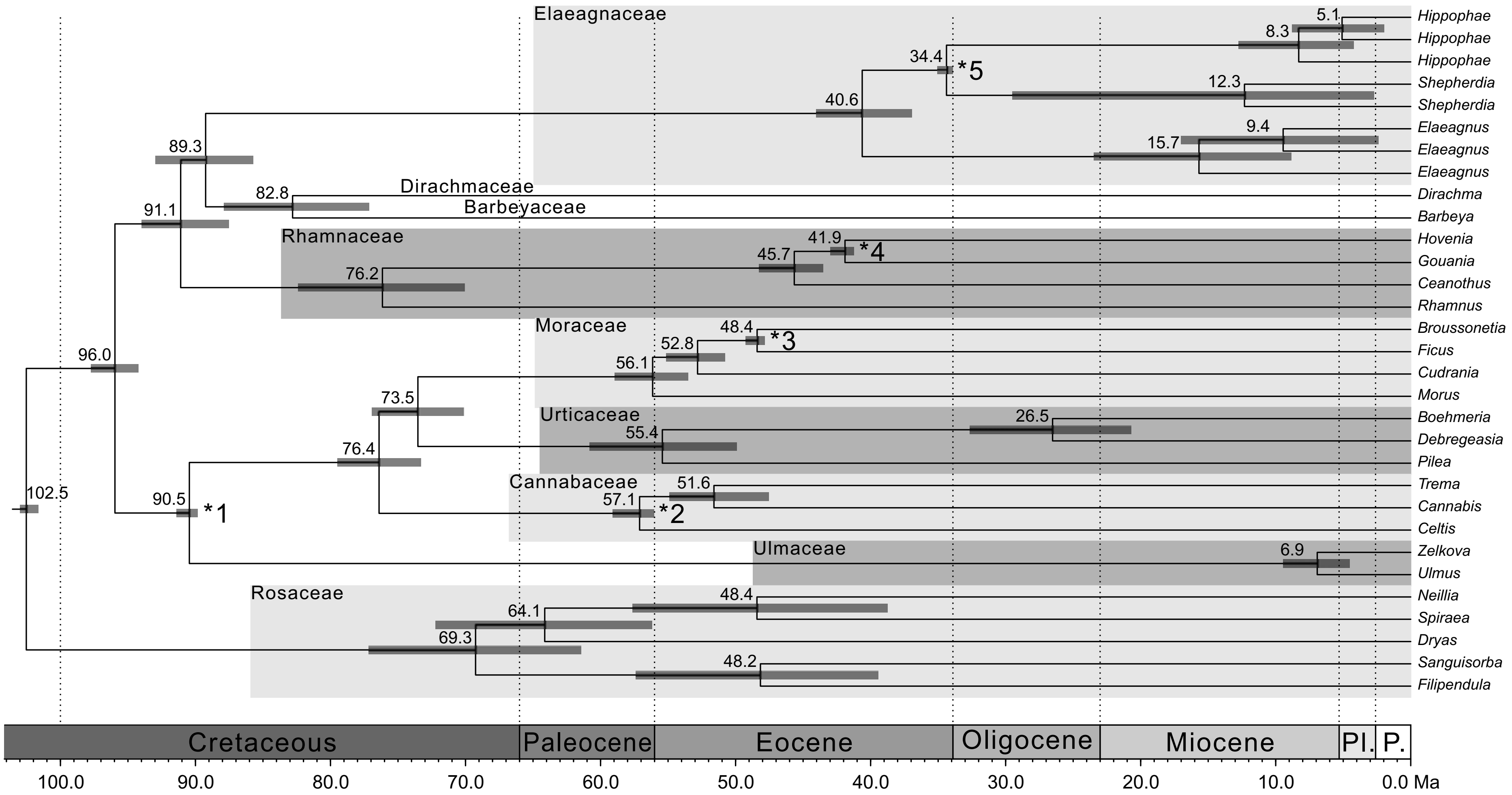

Figure S2. Chronogram of divergence times within Rosales derived from Bayesian relaxed clock analysis treating priors on fossils as being drawn from a lognormal distribution. Mean ages of nodes are shown, with horizontal dark grey bars indicating the 95% highest posterior density intervals. Fossil calibrations were used at nodes indicated with asterisks (see Table S4). Pl. = Pliocene; P. = Pleistocene.

References

Demesure, B., Sodzi, N., and Petit, R. (1995). A set of universal primers for amplification of polymorphic non-coding regions of mitochondrial and chloroplast DNA in plants. *Molecular Ecology* 4(1), 129-134.

Hamilton, M.B. (1999). Four primer pairs for the amplification of chloroplast intergenic regions with intraspecific variation. *Molecular Ecology* 8(3), 521-523.

Jia, D.R., Abbott, R.J., Liu, T.L., Mao, K.S., Bartish, I.V., and Liu, J.Q. (2012). Out of the Qinghai–Tibet Plateau: evidence for the origin and dispersal of Eurasian temperate plants from a phylogeographic study of *Hippophaë rhamnoides* (Elaeagnaceae). *New Phytologist* 194(4), 1123-1133.

Lewis, C.E., and Doyle, J.J. (2001). Phylogenetic utility of the nuclear gene malate synthase in the palm family (Arecaceae). *Molecular Phylogenetics and Evolution* 19(3), 409-420.

Li, M., Wunder, J., Bissoli, G., Scarponi, E., Gazzani, S., Barbaro, E., et al. (2008). Development of COS genes as universally amplifiable markers for phylogenetic reconstructions of closely related plant species. *Cladistics* 24(5), 727-745.

Sauquet, H., Ho, S.Y., Gandolfo, M.A., Jordan, G.J., Wilf, P., Cantrill, D.J., et al. (2012). Testing the impact of calibration on molecular divergence times using a fossil-rich group: the case of *Nothofagus* (Fagales). *Systematic Biology* 61(2), 289-313.

Shaw, J., Lickey, E.B., Beck, J.T., Farmer, S.B., Liu, W., Miller, J., et al. (2005). The tortoise and the hare II: relative utility of 21 noncoding chloroplast DNA sequences for phylogenetic analysis. *American Journal of Botany* 92(1), 142-166.

Strand, A., Leebens-Mack, J., and Milligan, B. (1997). Nuclear DNA-based markers for plant evolutionary biology. *Molecular Ecology* 6(2), 113-118.

Taberlet, P., Gielly, L., Pautou, G., and Bouvet, J. (1991). Universal primers for amplification of three non-coding regions of chloroplast DNA. *Plant Molecular Biology* 17(5), 1105-1109.

Walker, J.D., Geissman, J.W., Bowring, S.A., and Babcock, L.E. (2012). *Geologic Time Scale v. 4.0: Geological Society of America, doi: 10.1130/2012.CTS004R3C.*

White, T.J., Bruns, T., Lee, S., and Taylor, J.W. (1990). "Amplification and direct sequencing of fungal ribosomal RNA genes for phylogenetics," in *PCR Protocols: A Guide to Methods and Applications,* eds. M.A. Innis, D.H. Gelfand, J.J. Shinsky & T.J. White. (New York: Academic Press), 315-322.
